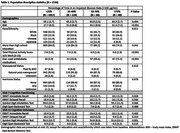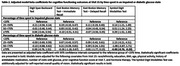# Time spent in an impaired glucose state across the midlife and early late adulthood cognitive function: Study of Women’s Health Across the Nation

**DOI:** 10.1002/alz.086154

**Published:** 2025-01-09

**Authors:** Aleda M. Leis, Carrie A. Karvonen‐Gutierrez, Rebecca C Thurston, Samar R. El Khoudary, Carol A. Derby

**Affiliations:** ^1^ University of Michigan, Ann Arbor, MI USA; ^2^ Department of Psychiatry, School of Medicine, University of Pittsburgh, Pittsburgh, PA USA; ^3^ University of Pittsburgh, Pittsburgh, PA USA; ^4^ Department of Neurology, and Department of Epidemiology and Population Health, Albert Einstein College of Medicine, Bronx, NY USA

## Abstract

**Background:**

Over 30 million Americans have diabetes, with 9 million likely undiagnosed. Diabetes is associated with cognitive decline and risk for Alzheimer’s disease and related dementia (ADRD). The lifetime impact of diabetes and prediabetes on cognition may be cumulative. We examined whether time spent in an impaired glucose state during midlife was associated with worse cognitive function in early older adulthood.

**Method:**

The Study of Women’s Health Across the Nation (SWAN) is a longitudinal multi‐center cohort of women followed from 42‐52 years. This cross‐sectional analysis includes 1336 women with cognitive testing at Visit 15, baseline fasting blood glucose measures, cognitive testing at Visit 7, and no history of myocardial infarction or stroke. Percentage of visits with an impaired glucose state (IGS; fasting glucose >100µg/mL), or diabetic state (DS; fasting glucose >126µg/mL), were considered, categorized as <25%, 25%‐<50%, 50%‐<75%, and ≥75%. Visit 15 outcomes included working memory (Digit Span Backwards (DSB) test), immediate and delayed memory recall (East Boston Memory Test (EBMT‐I and EBMT‐D)) and processing speed (Symbol Digit Modalities (SDMT) test). Separate linear regression models were constructed examining cumulative time in IGS or DS with each outcome at Visit 15, adjusting for Visit 15 covariates: race/ethnicity, education, BMI, age, physical activity, history of antidiabetic medication, number of visits with glucose, prior cognitive function score at Visit 7, and hormone therapy. SDMT models were adjusted for self‐reported overall quality of vision.

**Result:**

Women were mean age 65 years (SD:2.7) (**Table 1**) and 50% were non‐Hispanic White. The majority spent <25% of their visits in IGS or DS, and few spent ≥75% time in IGS or DS (6.7% and 0.9%, respectively). IGS was not associated with cognitive outcomes (**Table 2**). However, those with ≥75% time in DS had a 4.5 unit (95%CI:‐8.8,‐0.2;p = 0.040) lower SDMT score compared to those with <25% time in DS.

**Conclusion:**

While mild glucose impairment over midlife was not related to cognitive function in early older adulthood, prolonged diabetic‐level glucose was associated with worse processing speed in early older adulthood, suggesting the importance of diabetes screening and optimizing glucose control during midlife to preserve cognitive function at older ages.